# Activated zinc transporter ZIP7 as an indicator of anti-hormone resistance in breast cancer

**DOI:** 10.1039/c9mt00136k

**Published:** 2019-09-04

**Authors:** Silvia Ziliotto, Julia M. W. Gee, Ian O. Ellis, Andrew R. Green, Pauline Finlay, Anna Gobbato, Kathryn M. Taylor

**Affiliations:** a Breast Cancer Molecular Pharmacology Unit , School of Pharmacy and Pharmaceutical Sciences , Redwood Building , Cardiff University , King Edward VII Avenue , Cardiff , CF10 3NB , UK . Email: taylorkm@cardiff.ac.uk; b Nottingham Breast Cancer Research Centre , Division of Cancer and Stem Cells , School of Medicine , The University of Nottingham , Nottingham City Hospital , Nottingham , UK

## Abstract

ZIP7, a member of the ZIP family of zinc importers, resides on the endoplasmic reticulum membrane and transports zinc from intracellular stores to the cytoplasm after activation by CK2 phosphorylation on two serine residues (S275 and S276). ZIP7 is known to be required for the growth of anti-hormone resistant breast cancer models, especially those with acquired tamoxifen resistance developed from MCF-7. Using our new pS^275^S^276^ZIP7 antibody which only recognises activated ZIP7 (pZIP7), we have demonstrated that the hyperactivation of ZIP7 is prevalent in tamoxifen-resistant breast cancer cells. This evidence suggests that pZIP7 might have potential as a biomarker of acquired resistance to such anti-hormones in breast cancer, a current unmet clinical need. In this regard, we have also developed a new immunohistochemical assay for pZIP7 which allowed pZIP7 to be tested on a small clinical series of breast cancer tissues confirming its prevalence in such tumours and relationship to a variety of clinicopathological parameters and biomarkers previously associated with endocrine resistant phenotypes, notably increased activated MAPK signalling, expression of ErbB2, CD71 and the proto-oncogene c-Fos, as well as with increased tumour grade.

## Abstract 2

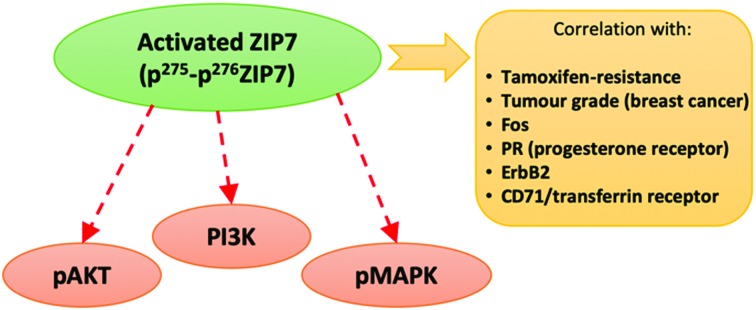
Zinc transporter ZIP7 activates key downstream signalling pathways and is correlated with important clinicopathological parameters that are associated with endocrine resistance.

##

Significance to metallomics

Our work elucidates the role of zinc in breast cancer and in particular its relevance to endocrine resistance, a currently unmet need in the therapy of oestrogen-positive breast cancer. While our previous studies have identified a rise in intracellular zinc as a characteristic typical of anti-hormone resistance, here we have expanded this research and identified the mechanism that leads to this event. This work increases our understanding of the role of zinc transporters in the development of diseases such as cancer, an aspect of zinc biology which still remains elusive.

## A Introduction

Zinc is one of the most important trace elements in the human body, acting as a cofactor for more than 300 enzymes.^1^ It is essential for several bodily functions including RNA transcription, DNA synthesis, cell division and activation of growth factors promoting signalling pathways.^2^ Recently, zinc deficiency and uncontrolled cellular zinc levels have been implicated in a number of important diseases,^3^ such as growth retardation,^4^,^5^ immunodeficiency,^6^ neurodegeneration,^7^ diabetes and cancer,^3^,^8^,^9^ making it a potential target for therapy. Furthermore, zinc excess is also associated with increased migration and exaggerated cell growth making zinc dysregulation an important driver of cancer.^9^ In particular, there is clinical evidence of increased zinc levels in breast cancer tissues when compared to normal breast tissue,^10^ suggesting the importance of maintaining proper zinc homeostasis in tissues. The first zinc transporter to be related to breast cancer was ZIP6 (also called SLC39A6 and LIV-1) which is an oestrogen-regulated gene^11^ associated with cancer spread to the lymph nodes^12^ and a feature of luminal A breast cancer.^13^

Zinc cannot traverse cell membranes and therefore relies on two families of zinc transporters, the ZnT family (termed SLC30A) of zinc exporters and the ZIP family (termed SLC39A) of zinc importers^14^ to control cellular zinc homeostasis. ZIP7, an SLC39A family member, is situated on the endoplasmic reticulum membrane^15^ and is essential for the release of zinc from intracellular stores. This ZIP7-mediated zinc release requires phosphorylation by protein kinase CK2 on two serine residues (S275 and S276) on the long intracellular loop of ZIP7 between TM III and TM IV.^16^ We have now developed a unique monoclonal antibody which binds ZIP7 only when phosphorylated on these two serine residues^17^ and have demonstrated that the mobilisation of zinc induced by the activation of ZIP7 is involved in regulating growth factor signalling of many pathways known to be responsible for aggressive cancer growth. This effect is directly due to the ability of released zinc to inhibit multiple tyrosine phosphatases, especially PTP1B.^18^ These data confirm previous observations showing ZIP7 abundance in tumours and additionally its position as one of the top 10% genes overexpressed in many poor prognostic cancer states.^9^

Breast cancer is the second most commonly diagnosed cancer worldwide and the most common among women,^19^ making it a serious worldwide issue that still needs to be tackled. Breast cancer can be broadly classified according to the expression of three main proteins: ER (oestrogen receptor), HER2 (human epidermal growth factor receptor 2) and PR (progesterone receptor).^20^ The most common type of breast cancer is oestrogen receptor positive breast cancer (ER+), which can be targeted with endocrine therapy such as aromatase inhibitors (AIs), SERMs (selective estrogen receptor modulators) or pure oestrogen receptor antagonists (SERD) that aim to reduce or entirely suppress, respectively, the action of the protein.^21^ According to NICE guidelines, standard treatment for breast cancer in postmenopausal disease includes oestrogen deprivation with the use of an aromatase inhibitor, or tamoxifen if AIs are not tolerated.^22^ The anti-oestrogen tamoxifen (a SERM) is used as a first-line treatment in premenopausal women, while the further antioestrogen Faslodex® (a pure antagonist)^21^ can be used in later stage disease and leads to the degradation of the oestrogen receptor.^23^,^24^ Recently, tamoxifen was also found to be a useful chemoprevention agent for women at high risk of breast cancer,^25^ and although the risks associated with its prolonged use were uncertain, more recent studies have demonstrated that prolonging tamoxifen therapy up to 10 years or more instead of the usual 5 years treatment is beneficial and can reduce both mortality and recurrence.^26^

Unfortunately, some patients treated with endocrine agents, including anti-oestrogens, develop resistance^27^ resulting in disease relapse and cancer recurrence during treatment,^28^ generating a requirement for new targets to allow additional novel therapies to control this more aggressive state. With this aim, we have previously developed unique anti-hormone resistant breast cancer models, both to tamoxifen^29^ and Faslodex®,^30^ that aim to mimic the clinical development of acquired resistance and that can help in the discovery of new potential targets. Interestingly, our tamoxifen-resistant MCF-7 derived breast cancer model (TamR) has both a higher level of available zinc^8^ and an increased gene expression of zinc transporter ZIP7.^31^ Removal of ZIP7 from TamR cells confirmed its role in activating epidermal growth factor receptor (EGFR) and insulin-like growth factor receptor (IGF-1R),^8^ both of which are known to drive the growth of these TamR cells.^32^

Here we have extended these previous investigations to examine whether monitoring the phosphorylation of ZIP7 provides a better indication of changes in zinc signalling pathway activation in the anti-hormone resistant breast cancer models. We demonstrated that while our tamoxifen-resistant cells have increased total ZIP7 protein expression, there is a larger increase in activated ZIP7, suggesting utilisation of zinc signalling pathways. We have confirmed this by detecting concurrent activation of AKT, also known as protein kinase B which is a serine-threonine kinase with a well-established role in cancer progression and cell survival.^33^ AKT is known to be activated by ZIP7-mediated zinc release^17^ which is especially elevated in the tamoxifen-resistant cells. Furthermore, we have observed that pZIP7 is present in all tamoxifen-resistant cells compared to only a small percentage of MCF-7 cells, suggesting ZIP7 activation is key to driving their anti-hormone resistant growth. We compared these results to Faslodex®-resistant cells and also examined how they alter over a more prolonged antihormone exposure time frame to more fully-reflect clinical resistance. We have also developed an immunostaining test able to detect pZIP7 in clinical samples and demonstrated activity of ZIP7 in most breast cancer samples comprising a small clinical series, with interesting clinicopathological and biomarker correlations suggesting ZIP7 prevalence in endocrine resistant tumours. We thus propose that pZIP7 is a potential indicator of anti-hormone resistance, especially tamoxifen resistance, which with further study may prove to have biomarker value for cancer patients treated with anti-hormones.

## B Materials and methods

### Cell culture and materials

Antihormone responsive MCF-7 cells (human breast adenocarcinoma cells), a gift from AstraZeneca (Macclesfield, UK) and derived anti-hormone resistant cell lines were cultured for experimentation in phenol-red free RPMI 1640 (Roswell Park Memorial Institute), 5% charcoal stripped steroid-depleted fetal calf serum, 200 mM l-glutamine and antibiotics (10 IU per ml penicillin, 10 μg ml^–1^ streptomycin and 2.5 μg ml^–1^ amphotericin B). The development of the tamoxifen resistant (TamR)^29^ and Faslodex® resistant (FasR)^30^ cell lines has been previously described (representing resistance developing over 8–10 months). Longer term resistant cell lines were also used that were developed after a more prolonged exposure (3 years) of these models to tamoxifen (TamRL) or Faslodex® (FasRL) respectively.^34^ Tissue culture media and constituents were obtained from Life Technologies Europe Ltd (Paisley, UK) and plasticware from Nunc (Roskile, Denmark). For Western blot or Immunofluorescence 1–5 × 10^5^ cells were seeded into 60 mm or 22 × 22 mm × 0.17 mm thick glass coverslips respectively. Cells were kept at 37 °C in a 5% CO_2_ atmosphere.

### Western blot

Samples for Western blot were prepared as previously described.^17^ Primary antibodies were diluted 1 : 1000 for anti pZIP7 (MABS1262, Merck Millipore, USA), total ZIP7 (19429-1-AP, ProteinTech™, USA), total AKT (#9272, Cell Signaling, USA), pSer^473^AKT (#9271, Cell Signaling, USA), total MAPK (#9102, Cell Signaling, USA) and phospho-MAPK (#9101, Cell Signaling, USA) and 1 : 10 000 for β-actin or GAPDH (A3854, G9295, Sigma Aldrich, USA). Quantification of Western blot results was performed by normalisation to β-actin or GAPDH values and using Alpha DigiDoc software 4.10 or ImageJ for Mac OS. Some gels were cut horizontally to enable multiple probing (Fig. 3A–B) thus making the same GAPDH band appropriate for both sets of results.

### Immunofluorescence

Cells were fixed with 3.7% formaldehyde (Sigma Aldrich) and processed as previously described.^15^ Cells were probed for 1 hour with primary antibody (anti-pZIP7 1/100, MABS1262, Merck Millipore, USA) followed by goat anti-mouse Alexa Fluor 594 secondary antibody at 1/1000 dilution (Molecular Probes, Invitrogen, UK) for 30 minutes. Coverslips were mounted on microscope slides with Vectorshield mounting medium with DAPI (Vector Laboratories, USA) and sealed with nail varnish. Cells were visualised on a Leica RPE automatic microscope using a 63× oil immersion lens. Pictures were acquired and processed using Openlab software for Macintosh operating system.

### Immunohistochemistry

All pellets of the cell models or breast cancer tissue samples used for the immunohistochemical assays had been formalin-fixed, embedded in paraffin, sectioned and mounted onto charged slides. The breast cancer series comprised *n* = 93 primary breast cancer samples who had presented for surgery at Nottingham City Hospital, with full ethical approval for immunohistochemical studies (Nottingham Research Ethics Committee REC2 C2020313). The sections were then washed in xylene and different concentrations of ethanol (from 100% to 70%) through to water in order to rehydrate the sample. After trying different antigen retrieval conditions, the optimal condition for pZIP7 staining was ascertained to be pressure-cook microwaving at 950 W in pH 8 EDTA buffer for 2 minutes. The assay used the mouse monoclonal pZIP7 antibody (MABS1262, Merck Millipore, USA) optimised to a 1/8000 dilution for the cell pellets and 1/800 dilution for breast cancer sections. The samples were first washed with PBS/Tween 0.02% and then covered with 0.18% hydrogen peroxidase to block endogenous peroxidases before blocking the sections with a serum-free blocking reagent (DAKO, UK) for 20 minutes. Using the optimal dilution, the pZIP7 antibody incubation was carried out in a humidity chamber for an hour at room temperature for the cell pellets, whereas the tissue samples were incubated overnight at 23 °C. The slides were then washed twice with PBS/Tween 0.02% and probed with secondary antibody (Mouse Envision labelled polymer-HPR #K4001, DAKO) for up to an hour followed by another two washes with TBS/Tween 0.05%. The protein of interest was then visualised using a DAB (3′-3′-diamobenzidine) chromogen-substrate solution (DAKO). Counterstaining was performed with methyl green 0.05% (aq.). The slides were then coverslipped using DPX Mountant and visualised on an Olympus BH-2 microscope. After determining a representative field for assessment, the pZIP7 immunostaining of the specimens was evaluated by consensus of two personnel, recording percentage positivity according to staining intensity category (*i.e.* 1, 2 and 3 corresponding to negative, weak, intermediate and strong staining, respectively). The HScore was then calculated as previously described using these data for each specimen.^35^

### Fluorescence-activated cell sorting (FACS analysis)

Zinc was measured using the cell permeant zinc-fluorescent dye Fluozin-3AM (Invitrogen) using a BD FACSVerse Flow Cytometer. Cells, 70–80% confluent on 35 mm dishes, were trypsinised and incubated at 37 °C with 5 μM fluozin-3 for 30 minutes, followed by a 30 minute incubation in fresh medium with no Fluozin-3. FACS results were analysed using FlowJo software.

### Kaplan–Meier plotter analysis

ZIP7 gene expression data in relation to breast cancer survival was assessed using publicly available online Kaplan–Meier analysis (www.kmplot.com).^36^ Kaplan–Meier plots were generated using Affymetrix microarray expression of ZIP7 mRNA (ID: 202667_s_at) in tumour samples from breast cancer patients that were restricted to oestrogen receptor positive breast cancers treated with tamoxifen only (*n* = 740) or with any endocrine treatment or chemotherapy (*n* = 2061).

### Statistical analysis

For the *in vitro* studies, statistical analysis was performed using analysis of variance (ANOVA) with Dunnet *post hoc*. The difference was considered significant when *p* < 0.05. Data were plotted with standard error of the mean (SEM) from at least *n* = 3/4 experiments. Statistical analysis of the pZIP7 HScores for the clinical sample series was evaluated using SPSS non-parametric statistical analyses. Spearman's correlation test examined the relationship between pZIP7 HScore and various biomarkers or signalling pathway elements that had previously been evaluated using either immunostaining or PCR in the series.^37^ Mann-Whitney analysis was also performed to analyse pZIP7 level according to available clinicopathological parameters, notably tumour size, tumour grade, menopausal status or patient age. Statistical analysis was carried out using IBM SPSS or GraphPad software.

## C Results and discussion

### Expression and activity of ZIP7 in tamoxifen and Faslodex® resistance

**Fig. 1:**
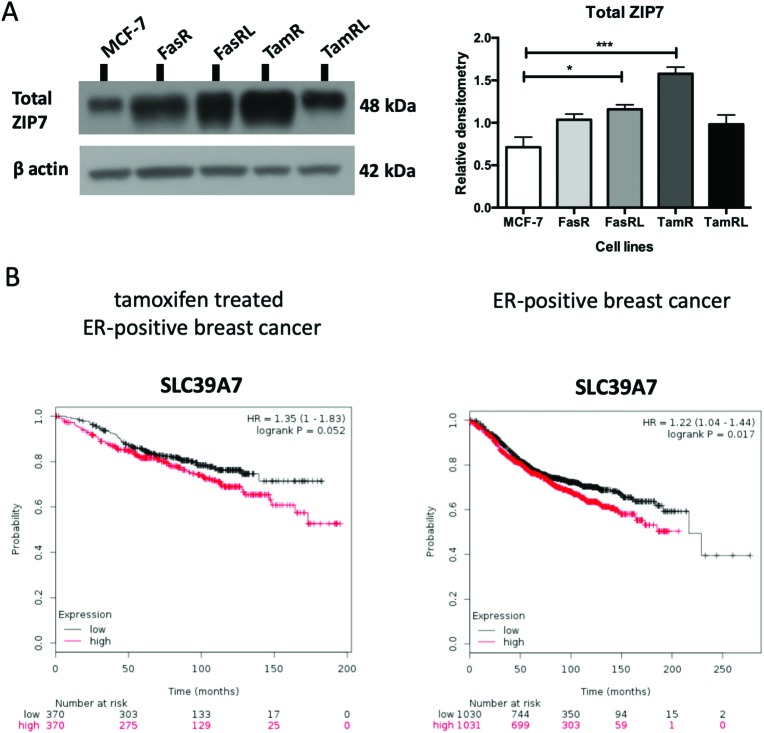
ZIP7 protein level is significantly increased in TamR and FasRL cells. (A) Immunoblotting of anti-hormone (tamoxifen or Faslodex®) resistant cell lines probed with total ZIP7 antibody and compared to MCF-7 cells. Statistical analysis was performed on densitometric data normalised to β-actin showing the mean values of *n* = 4 ± SEM with *** = *p* < 0.001 and * = *p* < 0.05 *versus* responsive MCF-7 cells. (B) KM plotter (; www.kmplot.com) derived survival plot showing patients with higher SLC39A7 mRNA expression had a shortened relapse free survival in both the tamoxifen treated ER+ (left graph; *n* = 740) and whole ER+ breast cancer patient cohorts treated why any endocrine treatment or chemotherapy (right graph, *n* = 2061).

Having previously seen an increase in ZIP7 expression and a role for its signalling in TamR cells^31^ we were keen to explore whether there was also a potential role for ZIP7 in further models of anti-hormone resistance. In order to examine whether changes in zinc signalling could be of more general importance in anti-hormone resistance, we thus first examined the protein expression levels of zinc transporter ZIP7 which is responsible for the release of zinc from intracellular stores. Using western blotting analysis, we compared the protein levels of ZIP7 in the four cell models of anti-hormone resistant breast cancer that had emerged by up to 3 years *in vitro*, mimicking clinical development of this disease state in many ER+ patients.^21^ These cell models aim to represent acquired resistance to shorter (TamR, FasR) and longer term (TamRL, FasRL) tamoxifen and Faslodex® treatment to better reflect adjuvant endocrine (antioestrogen) treatment used in breast cancer patients. A significant increase in total ZIP7 protein expression was detected in both the TamR (*p* < 0.001) and FasRL cells (*p* < 0.05) compared to MCF-7 cells (Fig. 1A). More modest increases were seen in the long-term TAMRL cells, which was slightly decreased compared with the shorter time of tamoxifen resistance. Data from the KM plotter tool showed that increased ZIP7 at the mRNA level is significantly associated with a shorter relapse-free survival (Fig. 1B) in ER-positive breast cancers, an association that is largely retained within the cohort of these patients treated with tamoxifen. These data fit with the results obtained with Western blotting, showing that a significant increase in total ZIP7 expression (SLC39A7) is also a feature typical of anti-hormone resistant breast cancer cells that appears particularly prominent for tamoxifen but also shows some increases in cells that have developed resistance to the further antioestrogen Faslodex® (Fig. 1A).

**Fig. 2:**
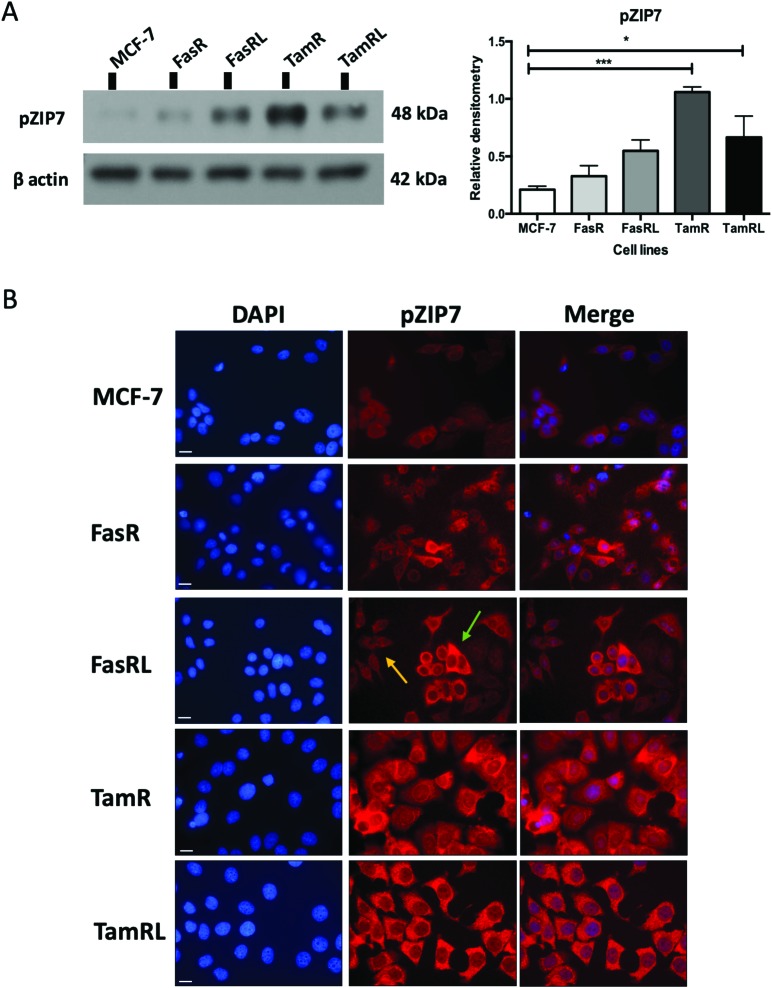
ZIP7 is significantly activated in TamR and TamRL cells. (A) Immunoblotting of anti-hormone (tamoxifen or Faslodex®) resistant cell lines probed with pZIP7 antibody and compared to MCF-7 cells. Statistical analysis was performed on densitometric data normalised to β-actin showing the mean values of *n* = 4 ± SEM with *** = *p* < 0.001 and * = *p* < 0.05 *versus* responsive MCF-7 cells. (B) Immunofluorescence of anti-hormone resistant cell lines probed for pZIP7 and compared to MCF-7 cells. Cells were seeded on coverslips, fixed with 3.7% formaldehyde and stained for pZIP7 antibody (red) along with DAPI (blue). Pictures are representative of multiple assessments of each cell line model (*n* = 3) imaged with a 63× oil immersion lens using a Leica Microscope. While staining is substantial in most cells for tamoxifen resistance, the arrows represent both highly pZIP7 positive (green) and lower positive (yellow) cells in the FasRL line. Scale bar: 10 μm.

Most importantly, using our pS^275^S^276^ZIP7 antibody we next revealed there was a significant four-fold increase in activated ZIP7 in the TamR cell line (*p* < 0.001) compared to MCF-7 cells (Fig. 2A). Moreover, there was a significant increase (*p* < 0.05) in pZIP7 in the long-term resistance model of TamR (TamRL), although with less amplitude, confirming the importance of ZIP7 signalling in driving tamoxifen resistance. While the Faslodex®-resistant cell line (FasR) showed no increase in activated ZIP7 compared to MCF-7 cells, interestingly there was a detectable increase in the level of pZIP7 as resistance progressed (FasRL) although this was more modest than seen with tamoxifen (Fig. 2A). These results in total suggested that levels of phosphorylated ZIP7 may be a valuable marker of anti-hormone resistance, particularly for tamoxifen, as there was evidence of active ZIP7-mediated zinc signalling in these cell lines.

### Assessing zinc signalling mechanisms in anti-hormone-resistant breast cancer cells

Having demonstrated a significant increase in ZIP7 activation in the TamR cells using Western blotting, we next wanted to examine whether this represented a moderate increase of activated ZIP7 in all cells or a large increase in a sub-population of the cells. Using fluorescent microscopy, we compared the staining for pZIP7 in the different cell lines using immunofluorescence. We observed that 80–100% of TamR and TamRL cells stained positively for pZIP7 (Fig. 2B) consistent with a location on the endoplasmic reticulum membrane as demonstrated in a previous study.^15^ We also observed much stronger staining in a proportion of the TamR cells, in accordance with the Western blotting showing particularly prominent ZIP7 activation in this model. In contrast, the FasR cells had a much lower percentage of pZIP7 positive cells that was very similar to the 10–20% positivity observed in the antihormone responsive MCF-7 cells. Interestingly, there were patches of FasRL cells that were more highly positive for pZIP7 surrounded by areas that were relatively pZIP7 negative (Fig. 2B, coloured arrows). This more obvious increase in the FasRL compared with FasR cells again reflected the Western blotting findings for pZIP7.

**Fig. 3:**
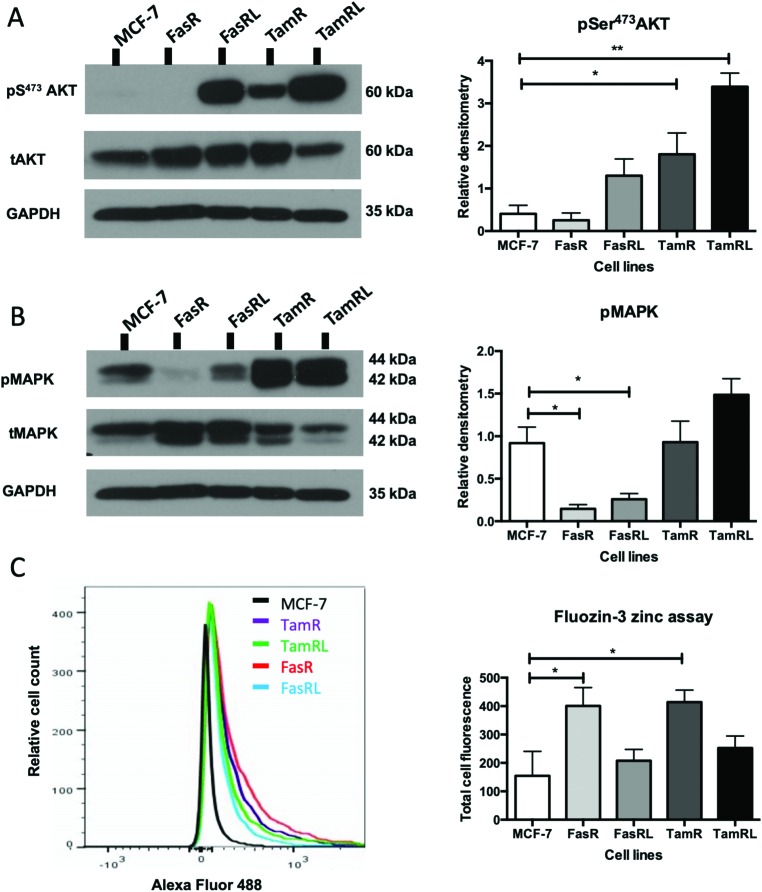
Activated ZIP7 increases intracellular zinc and activates AKT in anti-hormone resistance. Western blot comparing Faslodex® and tamoxifen anti-hormone resistant cell lines to MCF-7 cells probing for pS^473^AKT and total AKT (A) and pMAPK and total MAPK (B) on the same gel that had been cut horizontally thus making the same GAPDH bands appropriate for both parts. Statistical analysis was performed on densitometric data normalised to the total level of total AKT or MAPK showing the mean values of *n* = 4 ± SEM with ** = *p* < 0.01 and * = *p* < 0.05 *versus* responsive MCF-7 cells. The level of GAPDH is also shown. Zinc content of cells was imaged using the fluorescent zinc indicator Fluozin-3AM and the total fluorescence was documented using FACS analysis (C). Example plots are shown, with the graphical data showing mean values of *n* = 3± SEM with * = *p* < 0.05.

We have previously demonstrated that one of the major downstream targets of ZIP7-mediated zinc release^17^ is activation of AKT on residue S473, so we next used Western blotting to investigate whether activation of AKT was also observed in the cell models that exhibited higher pZIP7. Normalisation of phospho-AKT to the level of total AKT showed that the activation of AKT was considerably increased in TamR cells compared to the MCF-7 cells (*p* < 0.05) (Fig. 3A) which agreed with our previous data examining AKT signalling^8^ and was also consistent with the suggested role for zinc signalling in these cells. Interestingly, the longer-term variant of the TamR cells (TamRL) had an even greater increase in AKT activation (*p* < 0.01), suggesting further potential changes in zinc signalling with progression of tamoxifen resistance. While the Faslodex® resistant cell line (FasR) showed no increase in activated AKT, again there was a non-significant increase as resistance progressed in the FasRL cell line which was consistent with a potential change in zinc signalling in these cells (Fig. 3A). However, the same was not consistently observed with activation of MAPK in the MCF7-derived TamR line: while pMAPK was induced in some replicates, these cells showed no overall robust significant increase in MAPK activity compared to the MCF-7 cells, while the FasR cells showed a significant decrease (*p* < 0.05) (Fig. 3B). Furthermore, these levels did not significantly change in the long-term FasRL and TamRL resistant models compared with their earlier resistant counterparts.

### Intracellular zinc in anti-hormone resistant cell lines

Having demonstrated changes in ZIP7 and especially pZIP7 in these anti-hormone resistant cell lines we next assessed whether this corresponded to any measurable change in intracellular available zinc content. Cells loaded with the zinc-fluorescent dye Fluozin-3 had fluorescence assessed by FACS analysis and demonstrated an increased intracellular available zinc in TamR cells (*p* < 0.05) (Fig. 3C) which was consistent with that we had observed previously.^8^ Interestingly, the available zinc decreased somewhat from TamR cells to TamRL cells, consistent with the observed decrease in activated ZIP7 in the longer-term TamRL cells (Fig. 2B). Additionally, the FasR cells had significantly increased zinc levels (*p* < 0.05), similar to those seen for TamR cells (Fig. 3C) which also decreased with longer time of resistance. This interesting result seemed in conflict with the pZIP7 data but highlighted that deregulated zinc signalling may be a feature that is typical of anti-hormone resistant cells. One well described effect of ZIP7-mediated zinc release from stores is to inhibit multiple tyrosine phosphatases.^18^ This allows prolonged activation of tyrosine kinases such as EGFR and IGF1-R which are known to be responsible for driving the growth of TamR cells.^29^ One major target of intracellular zinc is PTP1B,^38^ a key phosphatase that dephosphorylates EGFR and IGF1-R, and has been shown to be inhibited by zinc at physiologically relevant concentrations such as picomolar.^39^

### Assessing ZIP7 activity in anti-hormone resistant cell lines using immunostaining

**Fig. 4:**
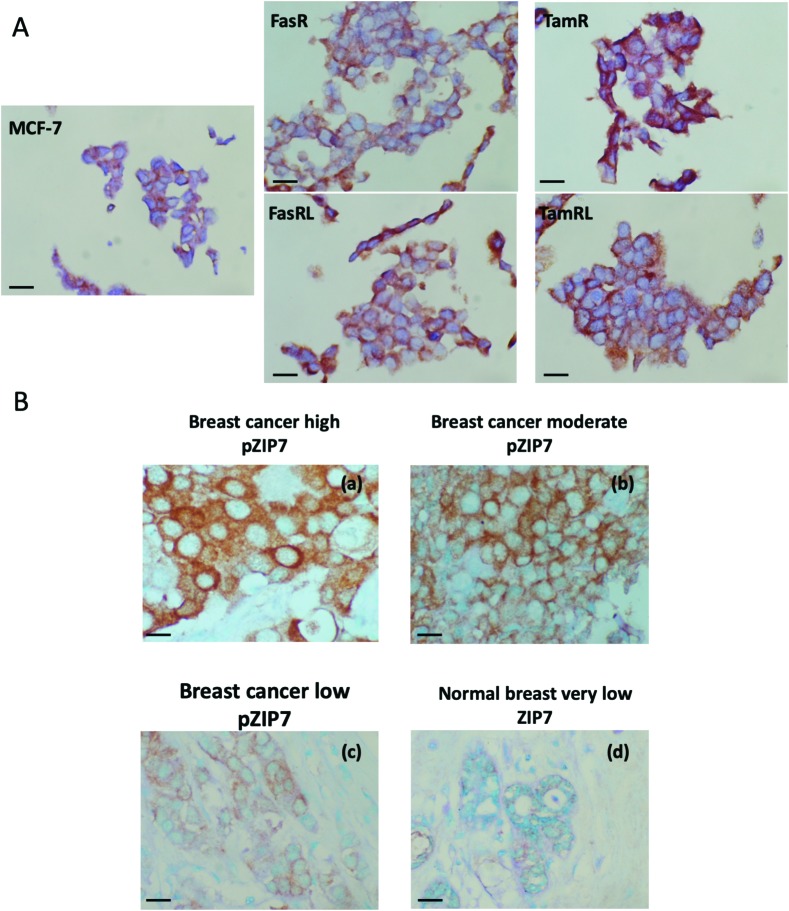
Immunohistochemical analysis of pZIP7 in breast cancer models and clinical samples. Formalin-fixed, paraffin-embedded cell pellet sections of Faslodex® and tamoxifen anti-hormone resistant cell lines and the responsive MCF-7 cells were immunostained with pZIP7 antibody using DAB (3′-3′-diaminobenzidine) chromogen, with methyl green counterstaining for the nuclei (A). Magnification: 40×. Scale bar: 25 μm. Immunostaining for pZIP7 in breast cancer sections from a clinical primary breast cancer series using the pZIP7 antibody (B). Samples are representative of high, moderate and low staining of pZIP7 in the series (a–c), all of which was greater than that seen in the tumour associated normal breast tissue also on the sections (d). pZIP7 was visualised using DAB chromogen staining, with methyl green counterstaining for the nuclei. Magnification: 40×. Scale bar: 25 μm.

We developed an immunohistochemical assay for our pZIP7 antibody (optimised using heat-mediated antigen retrieval) as we wished to test paraffin-embedded clinical breast cancer samples. We initially tested this method on cell pellets of our anti-hormone resistant cells to monitor if there was consistency with our Western blotting findings. The investigation on cell pellets of the five cell lines (Fig. 4A) revealed a strong staining for pZIP7 in the TamR cell line compared with MCF7, with all cells staining positively and some showing 3+ intensity staining, consistent with the TamR cells utilising zinc signalling pathways. Furthermore, the TamRL cells were also all positive for pZIP7 but the staining was not as strong as that seen for the TamR, again consistent with activation of zinc signalling at a lesser level than TamR as seen from the Western blotting and fluorescence studies. In contrast, the FasR cells showed more heterogeneous staining with a less prominent increase compared with the MCF-7 cells than seen for the tamoxifen resistant lines. Interestingly, the FasRL cells had some more positive cells for pZIP7 than the FasR cells, agreeing with the Western blotting and fluorescence observations (Fig. 2A and B). HScore evaluation of the cell pellet staining confirmed these results with TamR cells showing the highest pZIP7 HScore, confirming that this cell line has active utilisation of ZIP7-mediated zinc signalling (Table 1) and corroborating all previous data.

**Table 1:** HScore values of cell pellets. The table indicates the HScore for the pellet corresponding to each different resistant cell line in comparison to MCF-7 cells

Cell line	HScore
MCF-7	95
FasR	115
FasRL	160
TamR	220
TamRL	175

### Assessing ZIP7 activity in clinical breast cancer samples using immunostaining

In order to explore whether ZIP7 was also active in breast cancer patients and following on from the staining analysis of the cell pellets, we evaluated the pZIP7 antibody immunohistochemically in a clinical series of paraffin-embedded breast cancer sections obtained from Nottingham City Hospital. This series comprised samples from 93 primary breast cancer patients. Using the assay optimised with heat-mediated antigen retrieval, there was pZIP7 staining in all the tumour epithelial cells in the cancer tissues, demonstrating a widespread positivity to pZIP7 spanning a range of positive staining scores (Fig. 4B). pZIP7 staining was consistent with the protein location on the endoplasmic reticulum membrane. Although some of the cancer samples had lower levels of pZIP7 staining, there was still evidence of staining in virtually all cancer tissue (Fig. 4B), with an HScore of 0 in only one patient. The range of positivity was up to an HScore of 250, with a median HScore for pZIP7 staining in the series of 117. Interestingly, pZIP7 staining was only very low in tumour associated normal tissue (Fig. 4B), contrasting the widespread staining of pZIP7 in the breast cancers.

This clinical series had previously been assessed in some samples at the protein, phospho-protein or mRNA level for a variety of signalling parameters known to be relevant to endocrine resistance biology. While there were no outcome data associated with these samples it was possible to examine the levels of pZIP7 *versus* key clinicopathological endpoints that were available for many patients, notably patient age, menopausal status, tumour size, tumour grade, tumour stage and proliferation (Tables 2 and 3). Of these clinicopathological features, the only significant parameter associated with pZIP7 level was tumour grade (Table 2) where the level of pZIP7 was significantly enriched in the higher-grade tumours (*P* = 0.046). There was also a positive relationship between MAPK activity (pMAPK) and pZIP7 (*p* = 0.019), confirming our recent data^17^ that pMAPK was a downstream target of pZIP7.^17^ There was also a weak trend with PI3K expression, a signalling element known to impact on AKT signalling (*p* = 0.092). Furthermore, there was a modest positive correlation of pZIP7 in the series with expression of the tyrosine kinase receptor ErbB2 (*p* = 0.028) (Table 3). The analysis also showed a significant indirect correlation between pZIP7 and the progesterone receptor (PR) (*p* = 0.036) although no significant correlation of pZIP7 was seen with the oestrogen receptor (ER) in either ER+ or ER– disease. Both ErbB2 and pMAPK, low PR, and high tumour grade are all known to be features associated with endocrine resistance and disease progression in breast cancer,^17^,^28^ suggesting that higher levels of pZIP7 may be associated with resistant patients. Additionally, we noticed a significant positive relationship between CD71 (transferrin receptor) and pZIP7 levels (*p* = 0.017). The transferrin receptor can transport zinc into cells in addition to its transport of iron^40^,^41^ and upregulation of the transferrin receptor may partially explain the increased intracellular available zinc observed in anti-hormone resistant cell models. Higher levels of the transferrin receptor have also been linked to tamoxifen resistance.^42^ Moreover, the analysis revealed a significant correlation between the proto-oncogene c-Fos and pZIP7 (*p* = 0.007). The proto-oncogene c-Fos has again previously being associated with tamoxifen resistance.^43^

**Table 2:** Mann–Whitney analysis examining pZIP7 in relation to clinicopathological parameters. The analysis of this data used the clinicopathological parameters available on the SPSS database

Clinicopathological parameters	*p* Value	Number of patients	Comment
Menopausal status	*p* = 0.522	*n* = 82	Non-significant
Tumour grade (1/2 low-moderate *vs.* 3 high grade)	*p* = 0.046	*n* = 91	Significant (pZIP7 levels higher in grade 3 *vs.* 1/2)
Tumour stage (stage 1 = lymph node negative disease *vs.* 2/3 = lymph node positive disease)	*p* = 0.331	*n* = 73	Non-significant

**Table 3:** Spearman's correlation analysis between pZIP7 and different endocrine markers. The analysis used the biomarker data available on the SPSS database

Molecule	Test	Correlation	*p* and *R* values	Number	Comment
Steroid hormone receptors					
ER	Protein	No association	*p* = 0.809, *R* = –0.025	*n* = 92	Non-significant
PR	Protein	Indirect	*p* = 0.036, *R* = –0.234	*n* = 81	Significant

Growth factor receptors					
ErbB2	mRNA	Direct	*p* = 0.028, *R* = 0.344	*n* = 41	Significant
EGFR	mRNA	No association	*p* = 0.488, *R* = –0.122	*n* = 41	Non-significant
CD71/transferrin receptor	Protein	Direct	*p* = 0.015, *R* = 0.252	*n* = 93	Significant

Transcription factors and other intracellular kinases					
pMAPK	Phospho-protein	Direct	*p* = 0.019, *R* = 0.244	*n* = 93	Significant
Fos	Protein	Direct	*p* = 0.007, *R* = 0.280	*n* = 92	Significant
PI3K	mRNA	Direct	*p* = 0.092, *R* = 0.103	*n* = 45	Weak association

Clinicopathological parameters					
Age	Protein		*p* = 0.460, *R* = –0.079	*n* = 89	Non-significant
Tumour size	Protein		*p* = 0.139, *R* = –0.170	*n* = 77	Non-significant
Proliferation (Ki67)	Protein		*p* = 0.383, *R* = –0.093	*n* = 89	Non-significant

The immunostaining observations in total suggested that pZIP7 might (with further study) be relevant as a novel biomarker for the diagnosis of breast cancer. Moreover, it will now be interesting to expand this study further to assess pZIP7 levels in breast cancer samples that have anti-hormone treatment data associated with them to evaluate if elevated pZIP7 relates to endocrine resistance.

## D Conclusions

Despite the importance and requirement of zinc for human health, which has been known for the last 60 years,^44^ relatively little is known about the role of zinc in disease. The involvement of zinc in several pathologies is an important emerging area and now includes many cancers such as prostate cancer,^45^,^46^ liver and pancreatic cancers,^47^–^49^ lung cancer,^50^ breast cancer^51^,^52^ as well as providing promise for new cancer treatments.

The present study is focused on ZIP7, a member of the LIV-1 subfamily of ZIP transporters which is known to be relevant in many poor prognostic cancer states.^9^ Recently, knockdown of SLC39A7 has been shown to inhibit cell growth and induce apoptosis in human colorectal cancer cells.^53^ ZIP7 has also been demonstrated to drive a tamoxifen-resistant breast cancer model (TamR),^8^ where such resistance is an increasing problem in the breast cancer clinic.^28^ ZIP7 activates downstream tyrosine kinase pathways such as EGFR, IGF-1R and Src in such cells^8^ as a direct result of its ability to inhibit tyrosine phosphatases and activate MAPK, PI3K and mTOR pathways,^17^ which are consistent with activation of these kinases, known to drive the growth of tamoxifen-resistant breast cancer.^8^

Here we have demonstrated a significant increase in pZIP7 in TamR cells compared to MCF-7 cells which was a more pronounced increase than that detected using the total ZIP7 antibody. Increased ZIP7 activation was also seen with more prolonged exposure to tamoxifen in the longer-term model, suggesting some importance for activation of zinc signalling pathways in resistance irrespective of the duration of tamoxifen treatment. Interestingly, we also discovered that in contrast to MCF-7 cells almost all of the TamR cells were positive for pZIP7 suggesting they were actively using zinc signalling pathways controlled by ZIP7-mediated zinc release from stores. These results were confirmed by immunohistochemistry on cell pellets of the same resistant cell lines (an observation that also helped validate the antibody for use on paraffin-embedded material). Furthermore, this result was also evident in the breast cancer tissue samples, where pZIP7 was expressed in virtually all tumours and at elevated levels in those with features that were associated with resistance and progression, suggesting a potential clinical relevance for pZIP7 as a biomarker of cancer and also potentially of tamoxifen failure. This elevation of activated ZIP7 was further confirmed by the increased intracellular available zinc levels observed which was consistent with our previous data showing increased zinc in TamR cells^8^ in conjunction with a raised expression of ZIP7.^31^ We also showed how the increased level of activated ZIP7 is consistent with activation of AKT in the TamR cells, a major downstream target of ZIP7-mediated zinc signalling^17^ and a molecule with a known role in cancer progression.^33^

These findings are supportive of our hypothesis that a higher zinc signalling capability may lead to the development of resistance in ER+ breast cancer patients treated with tamoxifen. It is fascinating to see the full extent of cell positivity for pZIP7 in the TamR cells which is irrespective of duration of exposure to the drug, making pZIP7 a prominent characteristic that appears typical of tamoxifen-resistance.

Although there was no obvious increase of pZIP7 in FasR cells compared to MCF-7 cells, there was a modest increase of pZIP7 in the FasRL line, suggesting a potential value for pZIP7 as a biomarker relating to resistance emerging after more prolonged Faslodex® treatment. The modest increase of activated ZIP7 in the long-term FasRL model could have been a result of the increased protein level of ZIP7 in this cell line as confirmed by measurement with the total ZIP7 antibody. Further to our studies previously carried out only in TamR cells, we also demonstrated that the FasR cells have a significantly increased level of available zinc, as noted for TamR cells.^8^ Surprisingly this increased level of available zinc did not reflect the activation of ZIP7 which was only higher in the long term FasRL cells. Moreover, the models of Faslodex® resistant breast cancer did not show the same increased activation of downstream pathways which have been directly linked to ZIP7 activation, such as AKT (and to a lesser degree MAPK),^17^ in the TamR cells. Indeed, this study revealed that FasR cells, both shorter and longer-term, had a significant decrease of MAPK signalling. This result could be reflective of the fact that these Faslodex® resistant models are oestrogen receptor negative,^54^ due to the action of Faslodex® exposure which induces the loss of the ER protein,^23^ contrasting the maintenance of this receptor in the TamR line.^55^ There is already an established cross-talk mechanism between the oestrogen receptor and MAPK pathway activation in the TamR cells.^56^ A recent investigation of clinical samples from patients treated with Faslodex® confirmed a significant decrease of active MAPK signalling.^57^ While the present study provided novel information regarding use of zinc signalling in FasR cells, it also highlighted that Faslodex® resistance may be driven by zinc signalling mechanisms other than ZIP7, which are yet to be fully understood. Nevertheless, the increase of intracellular available zinc in both the FasR cells and tamoxifen resistant lines has confirmed that an increase of intracellular zinc is a characteristic likely to be typical of endocrine resistance.

While considerable further study is needed, the investigation of pZIP7 using immunostaining on clinical breast cancer samples here is certainly suggestive of the potential for using pZIP7 as a novel biomarker to help diagnose breast cancer. pZIP7 staining positivity was abundant in almost all patient samples from the clinical series used in this investigation, in contrast to the tumour associated normal breast tissue which stained poorly for pZIP7. ZIP7 activation was abundant in the clinical samples irrespective of their ER status, suggesting that it could be a useful biomarker of breast cancer that is not restricted to ER-positive disease. More interestingly, analysis of clinicopathological features revealed an association between increased activated ZIP7 and breast cancer patients with the poor prognostic feature of high grade. Furthermore, our study has confirmed the previous mechanistic links reported between pZIP7 and active MAPK signalling.^17^ There was also a trend with PI3K expression, confirming previous associations of pZIP7 with activation of PI3K/AKT signalling.^17^ Moreover, pZIP7 levels associated with transferrin receptor CD71 expression in the breast cancer samples, which is known to transport zinc as well as iron^41^ and so may also contribute to the increased cytoplasmic zinc seen in endocrine resistant cells since this receptor is also expressed in such models.^42^ Although there is no data concerning iron levels in endocrine resistant breast cancer, it will be important to consider this for future studies, especially in light of the important role of both iron and zinc in cell growth.^58^,^59^ Interestingly, the transferrin receptor has already been associated with an increase in proliferation which is a hallmark of aggressive cancer and in particular breast cancers that have developed resistance to tamoxifen,^42^ suggesting this association may in part be due to transferrin receptor influxing zinc into cells. Delivery of zinc *via* transferrin receptor and by ZIP7 activation may thus synergistically promote a more aggressive, endocrine resistant phenotype of breast cancer. In keeping with this, pZIP7 staining in the breast cancer series here was also significantly associated with high tumour grade, as well as with pMAPK which can be a feature of endocrine resistant tumours.^60^ There was also an indirect association between pZIP7 and the progesterone receptor (PR), highlighting the possibility that pZIP7 is enriched in tumours that are PR negative and often tumours that are more likely to be endocrine resistant as the progesterone receptor is a known marker of hormone responsiveness.^61^ Moreover, tumours that are PR negative have been associated with a higher risk of mortality in comparison to PR positive tumours.^62^ Our study has additionally revealed a significant correlation between pZIP7 and the ErbB2 receptor. It is well established from previous studies that tumours which overexpress the ErbB2 (HER2) receptor are more likely to be endocrine resistant due to the increased activation of growth factor pathways.^63^ Another important discovery here was the association of pZIP7 to the proto-oncogene c-Fos. This proto-oncogene is known to be associated with zinc status, as experiments have shown that human bronchial cells supplemented with zinc exhibit a two-fold increase of c-Fos mRNA in comparison to cells with a basal level of zinc.^64^ Therefore, the increased level of zinc (discovered here in endocrine resistant cells) as a direct result of ZIP7 activation could be one mechanism promoting c-Fos in breast cancer. This adds further evidence to our hypothesis that the zinc/pZIP7 mechanism is a key driver of disease progression since previous associations have been reported between c-Fos and endocrine resistance.^43^

In total, our experimental and clinical profiling evidence indicate that activation of ZIP7 may be associated with poorer prognosis in breast cancer and with the risk of developing endocrine resistance. Future studies of clinical samples are needed to explore pZIP7 staining *versus* endocrine outcome. Nevertheless, it was perhaps also reassuring that there was some association between shorter time to relapse in tamoxifen treated patients and ZIP7, albeit at the mRNA level, using KM Plotter. The discovery here that pZIP7 might have potential as a biomarker of anti-hormone resistant breast cancer also paves the way for further investigations of targeting this new mechanism to tackle the development of resistance. Knowing that ZIP7 activation on residues S275 and S276 is induced by CK2 phosphorylation,^16^ one approach might be to target ZIP7 signalling using CK2 inhibitors. A CK2 inhibitor (CX-4945, Silmitasertib), which is currently under investigation for its possible use on drug resistant cells,^65^ has shown promising preliminary results (Taylor *et al.*, unpublished) suggesting it can slow the growth of TamR cells. Moreover, this CK2 inhibitor is able to be internalised in resistant cells with a subsequent ability to induce cell death.^65^ CK2 is a protein kinase whose role in tumorigenesis is well established^66^ and for this reason the drug CX-4945 is currently in further cancer clinical trials, including in combination with chemotherapy for its use against cholangiocarcinoma (ClinicalTrials.Gov Identifier: NCT02128282) and also for the treatment of multiple myeloma (ClinicalTrials.Gov Identifier: NCT01199718). This drug also has potential to block cell proliferation in haematological tumours and in other cancer cell lines *in vitro*.^65^,^67^ The use of the CK2 inhibitor CX-4945 on tamoxifen-resistant cells will require further investigation, but the promising preliminary data in TamR cells suggests study in endocrine resistant disease may also be warranted.

In conclusion, we have demonstrated the potential of pZIP7 as a biomarker for breast cancer, with our cell model and clinical breast cancer findings indicating particular promise in the context of patients developing resistance to anti-hormonal agents, notably tamoxifen. Furthermore, since ZIP7 is activated by protein kinase CK2, our data is also suggestive that using a CK2 inhibitor, such as CX-4945, in conjunction with anti-hormones is worthy of exploration in the context of preventing the onset and progression of resistant disease states.

## Conflicts of interest

There are no conflicts to declare.
